# Sotrastaurin, a PKC inhibitor, attenuates RANKL‐induced bone resorption and attenuates osteochondral pathologies associated with the development of OA

**DOI:** 10.1111/jcmm.15404

**Published:** 2020-07-11

**Authors:** Cong Pang, Liangbao Wen, Haikuo Qin, Bikang Zhu, Xuanyuan Lu, Shixing Luo

**Affiliations:** ^1^ Department of Orthopedics The Ninth Affiliated Hospital of Guangxi Medical University Beihai China; ^2^ Department of Orthopedics Shaoxing People's Hospital (Shaoxing Hospital Zhejiang University School of Medicine) Shaoxing China; ^3^ Department of Neurosurgery The First Affiliated Hospital of Guangxi Medical University Nanning China

**Keywords:** osteoclast, PKCδ, sotrastaurin, subchondral bone, therapeutics

## Abstract

Osteoarthritis (OA) is a common degenerative disease that affects the musculoskeletal structure of the whole joint, which is characterized by progressive destruction of both articular cartilage and subchondral bone. Treatment of the bone pathologies, particularly osteoclast‐mediated subchondral bone loss in the early stages of OA, could prevent subsequent cartilage degeneration and progression of OA. In the present study, the PKC inhibitor, Sotrastaurin, was found to inhibit RANKL‐induced osteoclast formation in vitro in a dose‐ and time‐dependent manner. In particular, SO exerted its anti‐osteoclastic effect predominantly at the early stages of RANKL stimulation, suggesting inhibitory effects on precursor cell fusion. Using mature osteoclasts cultured on bovine bone discs, we showed that SO also exerts anti‐resorptive effects on mature osteoclasts bone resorptive function. Mechanistically, SO attenuates the early activation of the p38, ERK and JNK signalling pathways, leeding to impaired induction of crucial osteoclast transcription factors c‐Jun, c‐Fos and NFATc1. We also showed that SO treatment significantly inhibited the phosphorylation of PKCδ and MARCKS, an upstream regulator of cathepsin K secretion. Finally, in animal studies, SO significantly alleviates the osteochondral pathologies of subchondral bone destruction as well as articular cartilage degeneration following DMM‐induced OA, markedly improving OARSI scores. The reduced subchondral bone loss was associated with marked reductions in TRAP(+) osteoclasts in the subchondral bone tissue. Collectively, our data provide evidence for the protective effects of SO against OA by preventing aberrant subchondral bone and articular cartilage changes. Thus, SO demonstrates potential for further development as an alternative therapeutic option against OA.

## 1 INTRODUCTION

Osteoarthritis (OA) is the most prevalent form of arthritis with a worldwide incident rate of over 70% and can be considered a leading cause of global disability.^1^, ^2^ OA is a debilitating joint disease that causes pain, joint stiffness and limited joint motion.^3^, ^4^ For many years, OA was considered primarily a cartilage disorder, but with advancement in technology and numerous studies, it is now recognized as a ‘whole joint disease’. Due to the intimacy and interdependence of joint tissues, pathological changes in one joint tissue will ultimately compromise the structural integrity and function of other joint tissues. Pathological changes that occur at the osteochondral junction in OA include articular cartilage degeneration, subchondral bone loss and osteophyte formation.^5^, ^6^, ^7^, ^8^

Despite our knowledge of the osteochondral pathologies that occur during OA, the molecular underpinning that leads to these changes in OA is not fully understood; indeed, an enigma still remains as to whether articular degeneration precedes subchondral bone loss or vice versa. Mounting evidence suggest that increased osteoclast activity and high bone turnover rate leading to subchondral bone loss are an early instigator for subsequent cartilage degeneration in the development of OA.^6^, ^9^ Undeniably, the integrity of the articular cartilage depends on the underlying subchondral bone to function as a shock absorbers against high peak stresses.^10^ Animal studies have shown in the early stages of OA, marked reduction in subchondral bone thickness in the as a result of elevated osteoclast activity leading to loss of structural integrity of the osteochondral junction and promoting cartilage degeneration.^11^, ^12^ Similarly, decreased connectivity of subchondral bone trabeculae has been observed in patients with early‐stage OA which further reinforce the notion that subchondral bone loss is an early event that precedes cartilage damage and degeneration. These studies have provided compelling evidence for the pharmacological targeting of osteoclast bone resorptive activity in the early stages of OA for the treatment of OA.^13^

Osteoclasts are unique and highly specialized multinucleated giant cells that possess the remarkable capacity to degrade mineralized bone and cartilage matrix.^14^, ^15^ Osteoclasts achieve this via the secretion of acid and bone lytic proteases such as TRAP and cathepsin K into the resorption pit which dissolves and degrades the underlying bone matrix, respectively.^16^ For osteoclast to carry out bone resorption, extensive cytoskeletal polarization and formation of podosomal F‐actin ring are essential.^17^ This leads to the formation of the sealing zone and ruffled border membrane through which acid and proteolytic enzymes are released into the space between the osteoclasts and adjacent bone surface.^18^ Protein kinase Cs (PKCs) are a family of serine/threonine kinases that play important roles in various cellular processes including cell proliferation, differentiation and survival. Of the numerous members, PKCδ has been found to be required for osteoclast bone resorption and survival.^19^, ^20^ In particular, PKCδ was shown to be necessary for ruffled border formation and cathepsin K secretion.^20^ As such, targeting of PKCδ activity may offer potential therapeutic benefits in treating the bone pathologies associated with the development and progression of OA.

In this study, we examined the effects of Sotrastaurin (SO), a small molecular weight indolylmaleimide‐based immunosuppressant that selectively and potently inhibits PKC, in osteoclast formation and bone resorption in vitro and potential in vivo benefits in murine model of surgical destabilization of the medial meniscus (DMM)‐induced experimental OA. Here, we found that SO inhibited osteoclast formation and bone resorption in vitro via the suppression of RANKL‐induced activation of MAPK (p38, ERK and JNK) signalling cascades. Furthermore, these anti‐osteoclastic and anti‐resorptive effects of SO conveyed protective effects against DMM‐induced subchondral bone loss and cartilage degeneration significantly improving OA outcomes.

## 2 MATERIALS AND METHODS

### 2.1 Ethics statement

The current experiment was authorized by approved by Zhejiang University Institutional Animal Care and Use Committee (No.12951).

All procedures related to animal use are carried out in accordance with the guidelines of the National Institutes of Health (NIH) Guide for the Care and Use of Laboratory Animals, and the maximum humane care for animals according to the guidelines approbated by Zhejiang University Institutional Animal Care and Use Committee.

### 2.2 Reagents, media, and antibodies

The protein kinase C (PKC) inhibitor, Sotrastaurin (SO), was obtained from Selleckchem. Dimethyl sulphoxide (DMSO; ST038) was from Beyotime Institute of Biotechnology. SO was dissolved in DMSO at a stock concentration of 100 mmol/L and stored at −80°C. Foetal bovine serum (FBS), Minimum Essential Medium Eagle‐Alpha Modification (α‐MEM) and streptomycin/ penicillin were procured from Gibco (Thermo Fisher Scientific). Cell Counting Kit‐8 (CCK‐8) Cell Proliferation/Cytotoxicity Assay Kit was acquired from Dojindo Laboratories Co., Ltd. The Actin Cytoskeleton Staining Kit was purchased from MilliporeSigma. Recombinant murine macrophage colony‐stimulating factor (M‐CSF) and recombinant murine receptor activator of nuclear factor‐kappa B (NF‐κB) ligand (RANKL) were from PeproTech. Specific rabbit polyclonal antibodies against phospho(p)‐PKCδ, p‐MARCKS, p‐p38 and total p38, p‐ERK and total ERK, p‐JNK and total JNK, NFATc1/NFAT2, c‐Fos, c‐Jun, and β‐actin were procured from Cell Signaling Technology. All antibodies were used at a dilution of 1:1000.

### 2.3 Isolation of primary murine bone marrow‐derived macrophages

Primary murine bone marrow‐derived macrophages (BMMs) were extracted from the long bones of C56BL/6 mice as previously described.^21^ Briefly, BMMs were isolated by flushing the marrow of excised femurs and tibias of 6 to 8‐week‐old C57BL/6 mice. Extracted BMMs were cultured in α‐MEM containing 10% FBS and 1% streptomycin/penicillin (complete α‐MEM) supplemented with 30 ng/mL M‐CSF at 37°C and humidified atmosphere of 5% CO_2_. Upon confluence, adherent M‐CSF‐dependent BMMs were considered osteoclast precursor cells and used in subsequent downstream experiments.

### 2.4 In vitro cell viability/cytotoxicity assay

The cytotoxic effects of SO were assessed by the highly sensitive colorimetric CCK‐8 assay as per manufacturer's instructions. Briefly, BMMs seeded into 96‐well plate at a density of 8 × 10^3^ cells/well in complete α‐MEM medium supplemented with 30 ng/mL M‐CSF for 24 hours were pre‐treated without or with increasing concentrations (3.125, 6.25, 12.5, 25 or 50 μmol/L) of SO for 144 hours (6 days). At the end of the experimental period, cells were incubated with 10 μL of CCK‐8 reagent for 3 hours at 37°C after which the absorbance at wavelength of 450 nm (with 650 nm reference filter) for each condition were measured on an ELx800 Absorbance Microplate Reader (BioTek Instruments). The CCK‐8 assay is based on the dehydrogenase conversion of the highly water soluble tetrazolium salt, WST‐8, to a yellow‐colour formazan dye, which is thus directly proportional to the number of viable cells.

### 2.5 In vitro osteoclast differentiation assay

Bone marrow‐derived macrophages were seeded into 96‐well plate at density of 8 × 10^3 ^cells/well in complete α‐MEM supplemented with 30 ng/mL M‐CSF for 24 hours. To examine the dose‐dependent effect of SO on osteoclast formation, cells were then stimulated with 100 ng/mL RANKL without or with increasing concentrations (1.5625, 3.125 or 6.25 μmol/L) of SO for 6 days. To assess the time‐dependent effect of SO on osteoclast differentiation, cells were stimulated with RANKL without or with 6.25 μmol/L of SO from day 0 to day 2 (D0‐D2; early stage), day 2 to day 4 (D2 ‐ D4; middle stage) or day 4 to day 6 (D4 ‐ D6; late stage) of experimental period. Cells stimulated with RANKL only throughout the 6 day period in the absence of SO were used as controls. Media containing M‐CSF, RANKL and SO were replaced every other day until typical well‐spread multinucleated osteoclasts were observed in RANKL‐only treated controls (around 6 days). At this point, cells were briefly and gently washed with phosphate‐buffered saline (PBS), fixed in 4% paraformaldehyde (PFA) for 30 minutes and then stained for tartrate resistance acid phosphatase (TRAP) activity. The number of TRAP‐positive multinucleated osteoclasts with three or more nuclei and the average cell‐spread area were quantified using ImageJ software (NIH).

### 2.6 Podosomal actin cytoskeleton immunofluorescence

BMM‐derived osteoclasts were cultured as described above in the absence or presence of increasing concentrations (1.5625, 3.125 and 6.25 μmol/L) of SO. When mature well‐spread osteoclasts were observed in RANKL‐only treated controls (around 6 days), cells were fixed with 4% PFA for 30 minutes, washed briefly with PBS and then permeabilized with 0.1% Triton X‐100 for 5 minutes at room temperature. Cells were then incubated with Actin‐Stain 488 Phalloidin for 30 minutes in the dark at room temperature. The nuclei were counterstained with 4ʹ,6‐diamidino‐2‐phenylindole (DAPI) for 5 mintues in the dark at room temperature. Fluorescence images were captured with the Eclipse TS100 Inverted Fluorescence Microscope (Nikon Instruments; Tokyo Japan). The number and average size of podosomal actin belt were quantified using ImageJ software.

### 2.7 In vitro osteoclast‐mediated bone resorption assay

M‐CSF‐dependent BMMs were stimulated with RANKL only for 3‐4 days or until they form into small pre‐osteoclasts and then transferred in the same amount onto the size 100 μm of sterilized bovine bone discs (Rongzhi Haida Biotech Co., Ltd), in triplicate. After adherence, cells were treated with 100 ng/mL RANKL without or with increasing concentrations (1.5625, 3.125 or 6.25 μmol/L) of SO. Culture media was replaced every 2 days for further 6 days. At the end of the experimental period, cells were removed by mechanical agitation and processed for scanning electron microscopy imaging on a FEI Quanta 250 (Thermo Fisher Scientific). The average bone resorption area with respect to total bone disc area was quantified for each experimental condition using ImageJ software.

### 2.8 RNA extraction and real‐time quantitative PCR (qPCR) gene expression analyses

Real‐time qPCR was used to assess the effects of SO on the expression of osteoclast marker genes during osteoclast formation. M‐CSF‐dependent BMMs seeded onto 6‐well plates at a density of 3 × 10^5 ^cells/well in the complete α‐MEM were stimulated with 100 ng/mL RANKL without or with increasing concentrations (1.5625, 3.125 or 6.25 μmol/L) of SO for 6 days. Total RNA was then extracted from cultured cells using RNAiso Plus Total RNA Extraction Reagent (Takara Bio), and first‐strand complementary DNA (cDNA) synthesis was carried out using 1 μg of extracted total RNA and HiScript II Q RT SuperMix (Vazyme Biotech Co. Ltd) in accordance with manufacturer's protocols. The resulting cDNA was used as templates for SYBR Green‐based qPCR Master Mix reaction (Takara Bio) and analysed using a ABI Prism 7500 Fast Real‐Time PCR System and associated software (Thermo Fisher Scientific). PCR cycling conditions were as follows: initial denaturation at 95°C for 10 minutes; following 40 cycles of 95°C for 10 seconds, 60°C for 20 seconds and 72°C for 20 seconds (amplification); and a final extension at 72°C for 90 seconds. The following specific primer sequences were used: *GAPDH* (Forward: 5′‐AGG TCG GTG TGA ACG GAT TTG‐3′, and Reverse: 5′‐TGT AGA CCA TGT AGT TGA GGT CA‐3′); *ACP5/TRAP* (Forward: 5′‐TCC TGG CTC AAA AAG CAG TT‐3′, and Reverse: 5′‐ACA TAG CCC ACA CCG TTC TC‐3′); *NFATc1* (Forward: 5′‐CAG CTG CCG TCG CAC TCT GGT C‐3′, and Reverse: 5′‐CCC GGC TGC CTT CCG TCT CAT A‐3′); *c‐Fos* (Forward: 5′‐CCA GTC AAG AGC ATC AGC AA‐3′, and Reverse: 5′‐AAG TAG TGC AGC CCG GAG TA‐3′); *PKCδ* (Forward: 5′‐CAGCCTCAGGCCAAGGTGTT‐3′, and Reverse: 5′‐ AGACTGTTTGCAATCCACGTCCT‐3′); *DC‐STAMP* (Forward: 5′‐CTT GCA ACC TAA GGG CAA AG‐3′, and Reverse: 5′‐TCA ACA GCT CTG TCG TGA CC‐3′); and *CTSK* (Forward: 5′‐CTT CCA ATA CGT GCA GCA GA‐3′, and Reverse: 5′‐TCT TCA GGG CTT TCT CGT TC‐3′). Gene expressions were normalized to the housekeeping gene *GAPDH* using the 2^−∆∆CT^ method.

### 2.9 Protein extraction and Western blot analyses

To assess the effects of SO on early RANKL‐induced signalling events, M‐CSF‐dependent BMMs seeded in 6‐well plates at a density of 5 × 10^5 ^cells/well were pre‐treated with vehicle (α‐MEM) or 6.25 µmol/L SO for 1 hour. Subsequently, cells were then stimulated with 100 ng/mL RANKL for 5, 15, 30 or 60 minutes. To assess the effects of SO on late RANKL‐induced signalling cascades, M‐CSF‐dependent BMMs were stimulated with 100 ng/mL RANKL without or with 6.25 μmol/L SO for 1, 3 or 5 days. Unstimulated (no RANKL) and untreated (no SO) cells were used as a timepoint 0 (mock) control. At the end of the experimental period, cells were lysed on ice with RIPA lysis buffer supplemented with phosphatase and protease inhibitor cocktail (Sigma‐Aldrich). After 20 minutes incubation, cell lysates were cleared by centrifugation at 12 000 *g* for 15 minutes at 4°C. Supernatants were collected, and the concentration of total cellular proteins in each sample was quantified using the bicinchoninic acid (BCA) assay. Protein samples were mixed with sodium dodecyl sulphate‐polyacrylamide gel electrophoresis (SDS‐PAGE) sampling buffer and denatured by boiling for 5 minutes. A 30 μg amount of each protein sample was resolved by 10% SDS‐PAGE, and separated proteins were then electroblotted onto PVDF membranes (Bio‐Rad Laboratories) overnight at 4°C. Membranes were blocked in 5% skim milk in Tris‐buffered saline Tween‐20 (TBST; 50 mmol/L Tris, pH 7.6, 150 mmol/L NaCl, and 0.1% Tween‐20) for 1 hour at room temperature and then incubated with indicated primary antibodies overnight at 4°C with gentle agitation. Following extensive washes with TBST, membranes were incubated with the appropriate HRP‐conjugated secondary antibody for 2 hours at 4°C. Membranes were extensively washed and then incubated with BeyoECL Plus Enhanced Chemiluminescence reagent (Beyotime Institute of Biotechnology) for protein band development. Immunoblot images were acquired on a ImageQuant LAS 4000 Science Imaging System (Fujifilm). Densitometric analyses of the protein bands were performed with ImageLab software (Bio‐Rad).

### 2.10 Destabilization of the medial meniscus (DMM)‐induced experimental osteoarthritis (OA) model

All laboratory procedures related to animal use abided by the provisions of the National Institutes of Health (NIH) Guidelines for the Care and Use of Experimental Animals and were approved by the Animal Care and Use Committee of Zhejiang University (No. 12951). All mice were housed in specific pathogen‐free environment of 22‐24°C and 50%‐60% humidity with 12 hour light/dark cycle. Mice were given free access to standard rodent chow and water ad libitum. The surgical destabilization of the medial meniscus (DMM) closely mimics the clinical manifestations of meniscal injury and has become an important model tool for the study of OA. Thus, the DMM‐induced OA murine model was established to explore the potential therapeutic effect of SO on the progression of OA. A total of seventy‐two 8‐week‐old C57BL/6 mice, 36 females and 36 males, were acquired from the Shanghai Laboratory Animals Centre (SLAC) and raised to the age of three months. Mice were then randomly assigned to one of six groups according to sex (n = 6 mice each), including two groups of Sham‐operated (intragastric administration of PBS only), two groups of DMM surgery and two groups of DMM + SO treatment (intragastric administration of 10 mg/kg SO). For DMM surgery, mice were anaesthetized with chloral hydrate and then subjected to surgical transection of the medial meniscotibial ligament of the right knee as previous described.^22^ Sham mice received the same surgical procedure without the transection of the medial meniscotibial ligament. Two days post‐surgery, mice were intragastrically administered with SO (treatment groups) or equivalent volume of PBS (Sham and DMM groups only) every other day for 4 or 8 weeks. No adverse reactions or animal fatalaties were recorded during the treatment period, and all mice exhibited normal behavioural activity. Mice were killed at the respective endpoints and the knee joints were excised, cleaned of soft tissues, fixed in 4% PFA, and then processed for micro‐computed tomography (micro‐CT) and histological assessments.

### 2.11 Micro‐CT scanning and analysis

The excised whole right knee joints from each experimental group were scanned using a μCT100 high‐resolution cabinet cone beam micro‐CT scanner (Scanco Medical). Serial tomographic images were acquired at X‐ray voltage of 70 kV, current of 200 μA and isometric resolution of 20 μm. The subchondral bone below the tibial plateau area was selected as region of interest for three‐dimensional reconstruction and morphometric analyses of bone structural parameters including total tissue volume (TV, mm^3^), bone volume to tissue volume ratio (BV/TV), trabecular thickness (Tb.Th, mm) and trabecular spacing (Tb.Sp, mm).

### 2.12 Histological assessments

The scanned knee joints were decalcified in 10% EDTA (pH 7.4) for 21 days at 4°C and then embedded into paraffin blocks. Serial sections of 3 μm thick were cut and processed for TRAP and Safranin O‐Fast Green staining in accordance with standard laboratory protocols. Histomorphometric assessments of the number of TRAP(+) osteoclasts and osteoclast surface per bone surface (Oc.S/BS) were quantified using Image‐Pro Plus software (Media Cybernetics). Histological scoring of cartilage degeneration and severity of OA was conducted according to the Osteoarthritis Research Society International (OARSI) grading system.^23^

### 2.13 Statistical analyses

The data presented in this study are expressed as mean ± standard deviation of at least three times independently conducted experiments. Statistical difference was determined by Student's *t* test or one‐way ANOVA with LSD test using SPSS 19.0 software (IBM). A *P*‐value <.05 or unless otherwise indicated was considered statistically significant.

## 3 RESULTS

### 3.1 Sotrastaurin inhibits RANKL‐induced osteoclast formation in vitro

**FIGURE 1:**
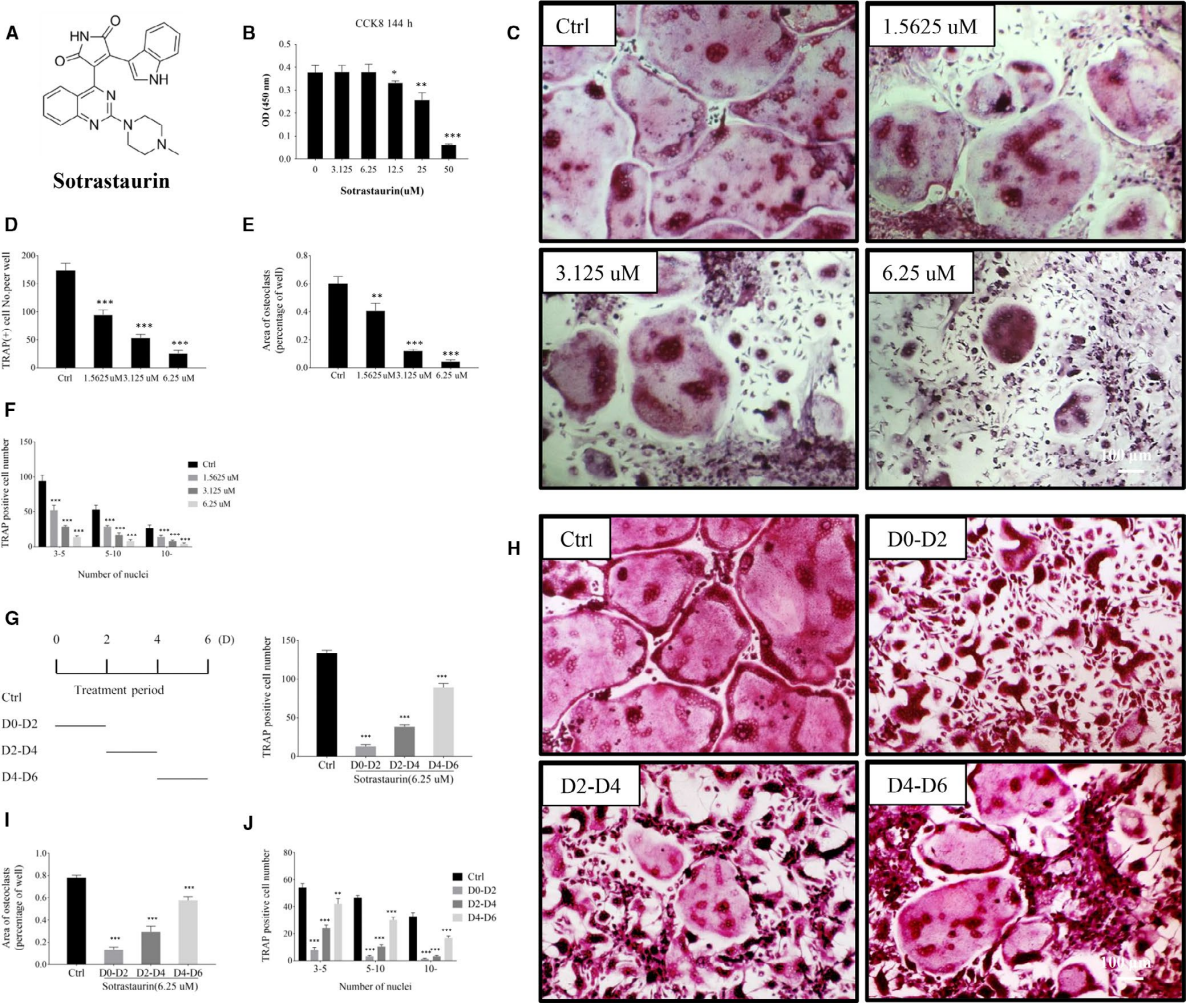
Sotrastaurin inhibited osteoclast formation induced by RANKL in vitro. A, The chemical structure of sotrastaurin. B, The effect of sotrastaurin on the activity of BMMs (bone marrow macrophages) in 144 h was assessed by CCK‐8 cell cytotoxicity/ proliferation test. C, Stimulation of M‐CSF (macrophage colony‐stimulating factor) dependent BMMS was performed with RANKL (receptor activator of nuclear factor‐κB ligand) for 6 d in the absence or presence of sotrastaurin in specified concentration, and subsequently fixated and stained for activity of TRAP. D‐F, Quantifiy the number and acreage of TRAP‐positive polynucleated osteoclasts (nuclei > 3). The amount and dimension of osteoclasts in each sotrastaurin concentration group were compared with the untreated controls. G and H, Sotrastaurin affected the osteoclastogenesis in a time‐dependent manner. BMMs (M‐CSF dependence) were stimulated with RANKL and treated with 6.25 μmol/L sotrastaurin within specified days, then fixated and stained to detect the activity of TRAP. G, I, J, Quantifiy the number and acreage of TRAP‐positive polynucleated osteoclasts (nuclei > 3). The amount and dimension of osteoclasts in each sotrastaurin concentration group were compared with the untreated controls. Data are expressed as means ± SD (*P < .05, **P < .01 and ***P < .001); SD, standard deviation. Scale bar, 100 μm. All experiments were performed at least three times

The potential cytotoxic effect of SO (Figure 1A, chemical structure) was first assessed using the CCK‐8 assay to identify sub‐lethal concentrations of SO for use in downstream cellular and biochemical assays. To this end, BMMs were treated with increasing concentrations (from 3.125 to 50 μmol/L) of SO for 6 days (144 hours), the typical length of time to generate osteoclasts from BMM precursor cells following RANKL stimulation. The results showed no inhibitory or cytotoxic effects of SO on BMM cell viability at concentrations below 6.25 μmol/L. However, a dose‐dependent and significant cytotoxic effect was observed at concentrations of 12.5, 25 and 50 μmol/L (Figure 1B). Thus, based on this result, SO concentrations of 1.5625, 3.125 and 6.25 μmol/L were selected for use in subsequent downstream cellular and biochemical assays.

We next examined the effects of SO on RANKL‐induced osteoclast formation. BMMs were stimulated with RANKL in the absence or presence of indicated concentrations of SO for 6 days to enable the differentiation and formation of multinucleated osteoclasts. As illustrated in Figure 1C, in the absence of SO, RANKL stimulation induced the formation of large well‐spread multinucleated osteoclasts that stained intensely for TRAP activity. However, when cells were cultured in the presence of SO, a dose‐dependent decrease in the total number of TRAP(+) osteoclasts formed was observed (Figure 1C,D). Additionally, the osteoclasts that formed following SO treatment were significantly smaller (Figure 1C,E) and less multinucleated (Figure 1F) than untreated controls, suggesting an inhibitory effect on BMM precursor cell fusion.

To further determine at which stage of osteoclast formation SO exhibit its inhibitory effect, we stimulated BMM cells with RANKL and then treated with 6.25 μmol/L SO on specified days of osteoclast formation. As shown in Figure 1G,H, the strongest inhibitory effect was observed when cells were exposed to SO early on in the RANKL‐induced osteoclast differentiation process between day 0 and day 2. Exposure of BMM cells within the first 2 days of RANKL stimulation markedly reduced their ability to differentiate and fuse into well‐spread multinucleated osteoclasts (Figure 1I,J). In contrast, when BMM cells were exposed to SO later in the differentiation process, that is between day 4 and day 6, only a moderate reduction in osteoclast formation was observed. Well‐spread TRAP(+) multinucleated osteoclasts were still present in the cultures. Treatment of cells with SO in the middle stage (between day 2 and day 4), the observed effects were in between that of early and late treatments (Figure 1G‐J).

**FIGURE 2:**
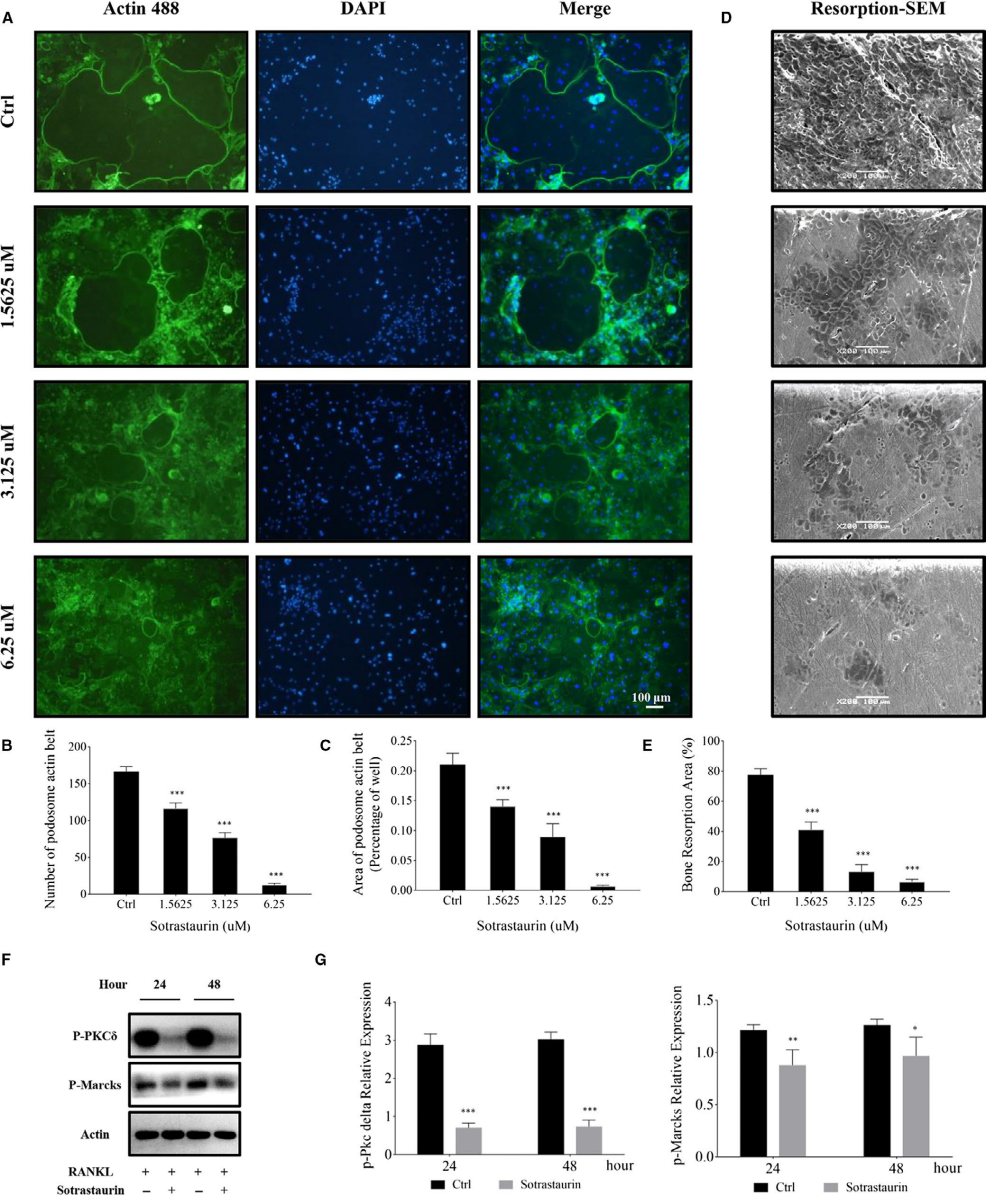
Sotrastaurin inhibits podosome actin belt formation and impairs osteoclasts mediated bone resorption in vitro. A, Effect of sotrastaurin on the formation of podosome actin belt. M‐CSF‐dependent BMMS were seeded onto 96 well plates and stimulated with RANKL without or with the specified concentration of sotrastaurin (0, 1.625, 3.125 and 6.25 μmol/L) for 6 d. Cells were fixed and stained for immunofluorescence. Typical fluorescence images of osteoclasts’ podosome actin belts (green) and nuclei (blue, DAPI). B and C, The number and area of podosome actin belts were quantified. Scale bar, 100 μm. D, Effect of sotrastaurin on osteoclast bone resorption activity. BMM‐derived pre‐osteoclasts were cultured on bone slices and stimulated with RANKL (100 ng/mL) in the absence or presence of increasing concentrations of sotrastaurin for 6 d. The cells were removed by ultrasound and the resorption pits were evaluated under a scanning electron microscope. Scale bar, 100 μm. E, Relative to the untreated controls, area of bone resorption pits under each treatment concentration was quantified. F, BMMs were treated with 6.25 μmol/L sotrastaurin, cells were incubated for 24 and 48 h and then harvested. The expression levels of phosphorylated PCKδ and MARCKS in protein lysates were analysed by Western blot. Expression of β‐Actin was analysed as an internal loading control. G, Densitometric analyses of the expression of the phosphorylation‐PKCδ and phosphorylation‐MARCKS were reported as p‐PKCδ/ β‐Actin and p‐MARCKS/ β‐Actin. Data are presented as the mean ± SD (*P < .05, **P < .01 and ***P < .001); SD: standard deviation. DAPI: 6‐diamidino‐2‐phenylindole

Osteoclast precursor cell fusion and multinucleation require significant reorganization of the cells cytoskeleton. An important and defining characteristic of well‐spread multinucleated osteoclasts is the formation of a F‐actin‐rich podosomal belt that circumscribes the cell. As illustrated in the immunofluorescence images in Figure 2A, osteoclasts from RANKL‐only treated BMM precursor cells formed well‐defined podosomal actin belts. On the contrary, BMM cells treated with SO showed a dose‐dependent inhibition in podosomal actin belt formation with significant reduction in overall size (Figure 2A‐C). In addition, compared to the control group, BMMs treated with higher concentrations of sotrastaurin predominantly remained as mononuclear monocytic cells rather than fused into multinucleated osteoclasts (Figure 2A). Taken together, these results provide compelling evidence that SO inhibits osteoclast formation in vitro by impairing precursor cell fusion.

### 3.2 Sotrastaurin impairs bone resorption in vitro

Having now shown that SO inhibits RANKL‐induced osteoclast formation via the impairment of precursor cell fusion, we sought to determine the effects of SO on mature osteoclast bone resorptive function. Given that SO inhibits the formation of the podosomal actin belt which is also required for bone resorption, we hypothesize that SO will inhibit mature osteoclast bone resorption in vitro. To this end, BMM‐derived osteoclasts cultured on bovine discs were stimulated with indicated concentrations of SO for 6 days after which cells were removed and resorption pits analysed. As shown in the scanning electron micrographs in Figure 2D,E, osteoclasts cultured without SO treatment displayed significant bone resorptive activity, resorbing close to 80% of the total bone disc area. On the other hand, osteoclasts treated with SO showed marked reductions in bone resorption capability (Figure 2D), resorbing only 40%, 10% or 5% of total bone disc area when treated with 1.5625, 3.125 or 6.25 μmol/L SO, respectively (Figure 2E). Previous studies have shown that PKCδ regulates cathepsin K secretion during osteoclast bone resorption via the modulation of myristoylated alanine‐rich C‐kinase substrate (MARCKS).^20^ Here, we further confirmed that the inhibition of osteoclast bone resorption activity by SO was due to inhibition of PKCδ and MARCKS phosphorylation (Figure 2F,G). Thus, these results further show that SO also exhibits anti‐resorptive effects against mature osteoclast bone resorption in vitro.

### 3.3 Sotrastaurin attenuated RANKL‐induced activation of MAPKs signalling cascade

**FIGURE 3:**
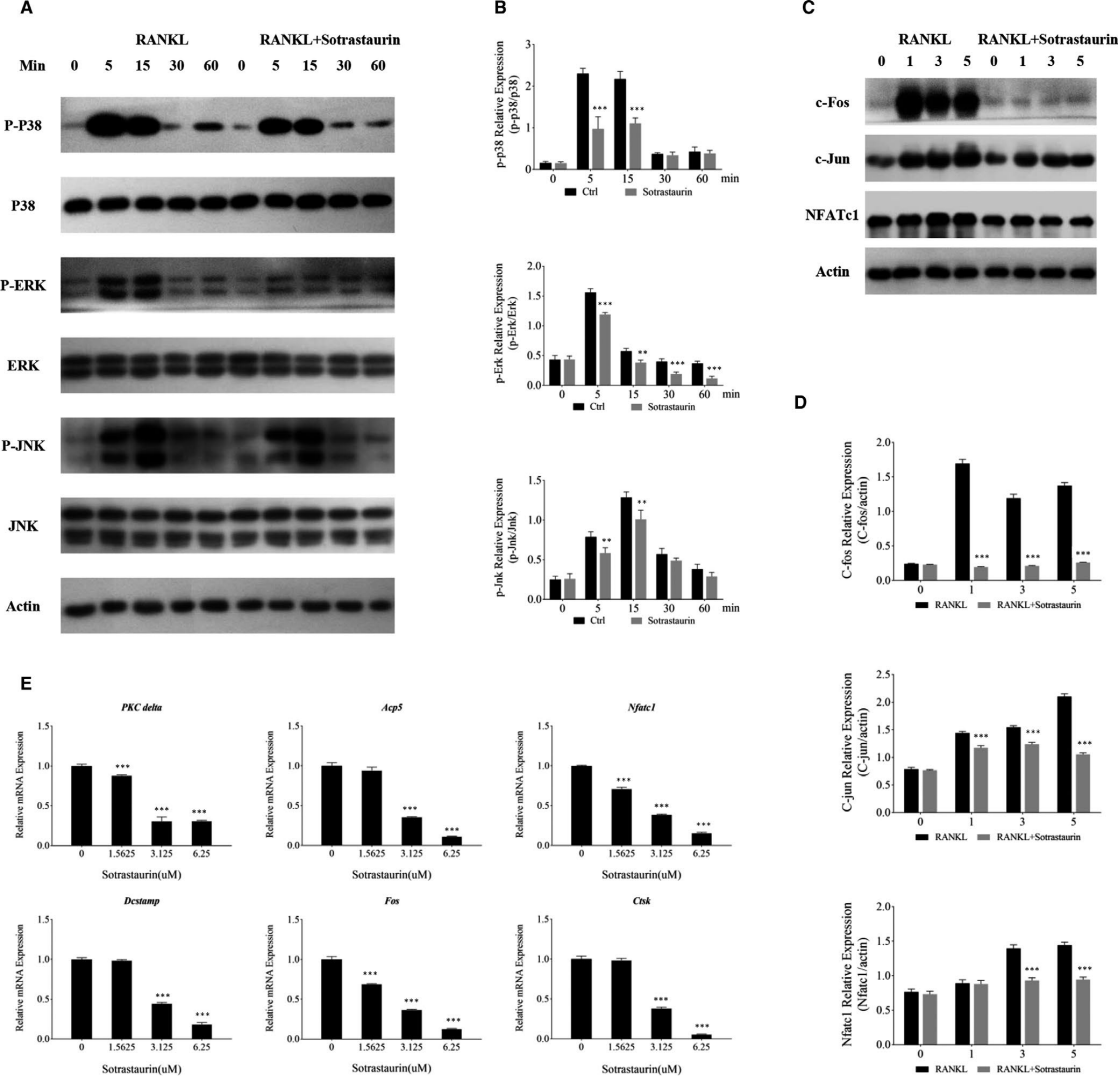
Sotrastaurin exerts its anti‐osteoclast effect by the inhibition of MAPKs signalling cascade. A, Sotrastaurin inhibited the activation phosphorylation of p38, JNK and ERK induced by RANKL. After pre‐treatment with 6.25 μmol/L sotrastaurin for 1 h, and then stimulated with RANKL for a specified time, Western blot analysis was performed with specific antibodies to mitogen‐activated protein kinases (p38, ERK and JNK) signalling cascade. β‐actin was used to act as an internal loading control. B, Relative change in the phosphorylation status of p38, JNK and ERK were determined by optical density analysis of each phosphorylated band, and each one was expressed as the ratio of its total protein counterpart. C, Sotrastaurin attenuated the expression of c‐Fos, c‐Jun and NFATc1 induced by RANKL. The total protein extracts of BMMs cultured in the medium containing RANKL and 6.25 μmol/L sotrastaurin for 0, 1, 3 and 5 d were analysed by Western blotting and specific antibodies to c‐Fos, c‐Jun and NFATc1. β‐actin was used to act as an internal loading control. D, The changes in protein expression level after sotrastaurin treatment were quantified by relative β‐actin density analysis and expressed as the ratio compared with the untreated group stimulated by RANKL alone. E, Sotrastaurin inhibited osteoclast marker genes induced by RANKL in a dose‐dependent manner. Real‐time quantitative PCR assessed the expression of *PKCδ, ACP5, NFATc1, DC‐STAMP, c‐Fos* and *CTSK* in osteoclasts treated with specified concentrations of sotrastaurin. The expression of the target genes were standardized with reference to the housekeeping gene *GADPH* and then displayed a decreased change compared with the untreated control groups. Data are presented as the mean ± SD (*P < .05, **P < .01 and ***P < .001); SD, standard deviation. MARCKs, myristoylated alanine‐rich C‐kinase substrate

The MAPK signalling pathway consisting of members, p38, ERK and JNK, is intimately involved in OA‐related cartilage destruction as well as osteoclast formation and bone resorption.^24^, ^25^, ^26^ Furthermore, PKCδ has been shown to be involved in the upstream regulation of MAPK signalling in response to RANKL‐RANK activation.^19^ Thus, we further investigated the effects of SO on RANKL‐induced signalling events. Binding of RANLK to receptor RANK rapidly induces the activation phosphorylation of all three members of MAPK signalling cascade. As shown in Figure 3A,B, p38, ERK and JNK phosphorylation was observed within 5 minutes of RANKL stimulation and lasting for around 15 minutes before returning to basal levels. In contrast, pre‐treatment of cells with SO significantly attenuated the activation phosphorylation of all three members of the MAPK signalling cascade (Figure 3A,B). The early activation of MAPK signalling pathways is required for the subsequent induction and activation of downstream nuclear transcription factors such as c‐Jun, c‐Fos and NFATc1. These nuclear factors particularly NFATc1 are crucial for the transcriptional regulation of effector genes necessary for efficient osteoclast differentiation, fusion and bone resorptive function. Here, we further showed that treatment of cells with SO abolished the induction of c‐Fos and markedly reduced the induction of c‐Jun and NFATc1 (Figure 3C,D).

As mentioned earlier, the induction of the nuclear transcription factors is necessary for the up‐regulation of osteoclast effector genes. These effector genes play essential roles in various stages of osteoclast differentiation, precursor cell fusion and mature osteoclast bone resorption. Using real‐time quantitative PCR analysis (Figure 3E), we showed that SO treatment dose‐dependently down‐regulated the gene expression of c‐Fos and NFATc1 transcription factors themselves which can account for their reduced protein induction. The gene expression of DC‐STAMP which is involved precursor cell fusion was similarly down‐regulated. Finally, the expression of genes encoding *PKCδ* and proteolytic enzymes required for bone resorption such as *ACP5/TRAP* and cathepsin K (*CTSK*) were also found to be reduced. Taken together, our biochemical immunoblot and qPCR analyses indicate that the anti‐osteoclastic effect of SO is, in part, due to the attenuation of RANKL‐induced MAPK activation and the downstream induction of nuclear transcription factors c‐Fos, c‐Jun and NFATc1 which consequently down‐regulated the expression of crucial osteoclast effector genes required for precursor differentiation and fusion, and mature osteoclast bone resorption.

### 3.4 Sotrastaurin treatment averts subchondral bone deterioration and articular cartilage degeneration in mice with DMM‐induced OA

**FIGURE 4:**
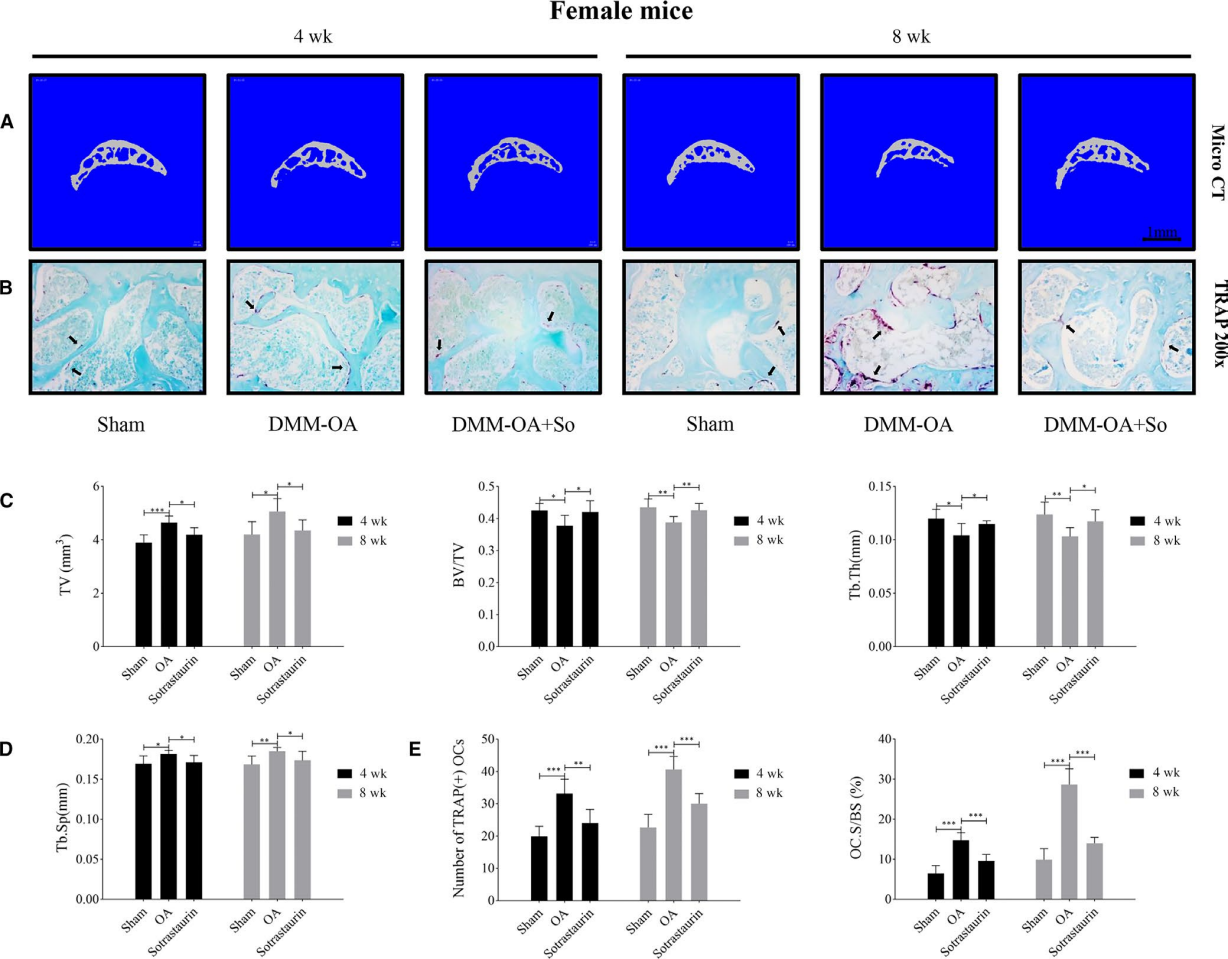
Sotrastaurin protects against subchondral bone loss in vivo after destabilization of the medial meniscus. A, Representative three‐dimensional micro‐CT images of the sagittal views of the medial compartment of tibial subchondral bone from Sham (Sham + PBS), OA (DMM + PBS) or Sotrastaurin (DMM + Sotrastaurin) groups at 4 and 8 weeks after operation. Scale bar, 1 mm. B, Representative images of knee joint subchondral bone were stained by fast green and osteoclasts in subchondral bone were stained by tartrate‐resistant acid phosphatase (TRAP) at 4 and 8 weeks after surgery (magnification, 200×); Black arrow indicates TRAP‐positive cells. C and D, Histogram of three‐dimensional structural parameters of tibial subchondral bone: (TV) tissue volume, (BV/TV) trabecular bone volume/tissue volume, (Tb.Th) trabecular thickness, and (Tb.Sp) trabecular separation. E, The number and area percentage of osteoclasts stained by TRAP in sections were measured. n = 6 per group. Data are expressed as means ± SD (*P < .05, **P < .01 and ***P < .001); SD, standard deviation

Given the encouraging anti‐osteoclastic and anti‐resorptive effects of SO, we next explore the potential in vivo therapeutic effects of SO against experimental OA (osteoarthritis) induced by surgical destabilization of the medial meniscus (DMM). DMM surgery in mice was performed on the right knee by transecting the medial meniscotibial ligament to induce cartilage degeneration and development of OA. Mice were then treated without or with SO for 4 and 8 weeks after which mice were killed, and subchondral bone deterioration and articular cartilage degeneration in the right knee joints were analysed by micro‐CT and histology respectively. Three‐dimensional reconstructions of the micro‐CT images showed significant subchondral bone loss in the DMM‐OA group when compared with Sham controls at both 4 and 8 weeks. In contrast, intragastric injections of SO mitigated the tibial subchondral bone loss induced by DMM surgery at both time points (Figure 4A, female mice; and Figure S1a, male mice). Consistent with increased subchondral bone deterioration, TRAP staining of tibial bone sections revealed significant elevations in the number of TRAP(+) multinucleated cells on the subchondral trabecular bone surface in DMM‐OA group as compared to Sham controls. Again, significantly less TRAP(+) multinucleated cells were noted following SO treatment (Figure 4B, female mice; and Figure S1b, male mice). Quantitative morphometric measurement of total tissue volume (TV, mm^3^), ratio of bone volume to tissue volume (BV/TV), trabecular thickness (Tb.Th, mm), trabecular spacing (Tb.Sp, mm), number of TRAP(+) osteoclasts and percentage osteoclast surface per bone surface (OC.S/BS, %) further confirmed the protective effects of SO against osteoclast‐mediated tibial subchondral bone loss following DMM surgery (Figure 4C‐E, female mice; and Figure S1c‐e, male mice). These results strongly indicate that SO exhibits potent inhibitory effects against osteoclast formation and osteoclast‐mediated bone destruction in vivo.

**FIGURE 5:**
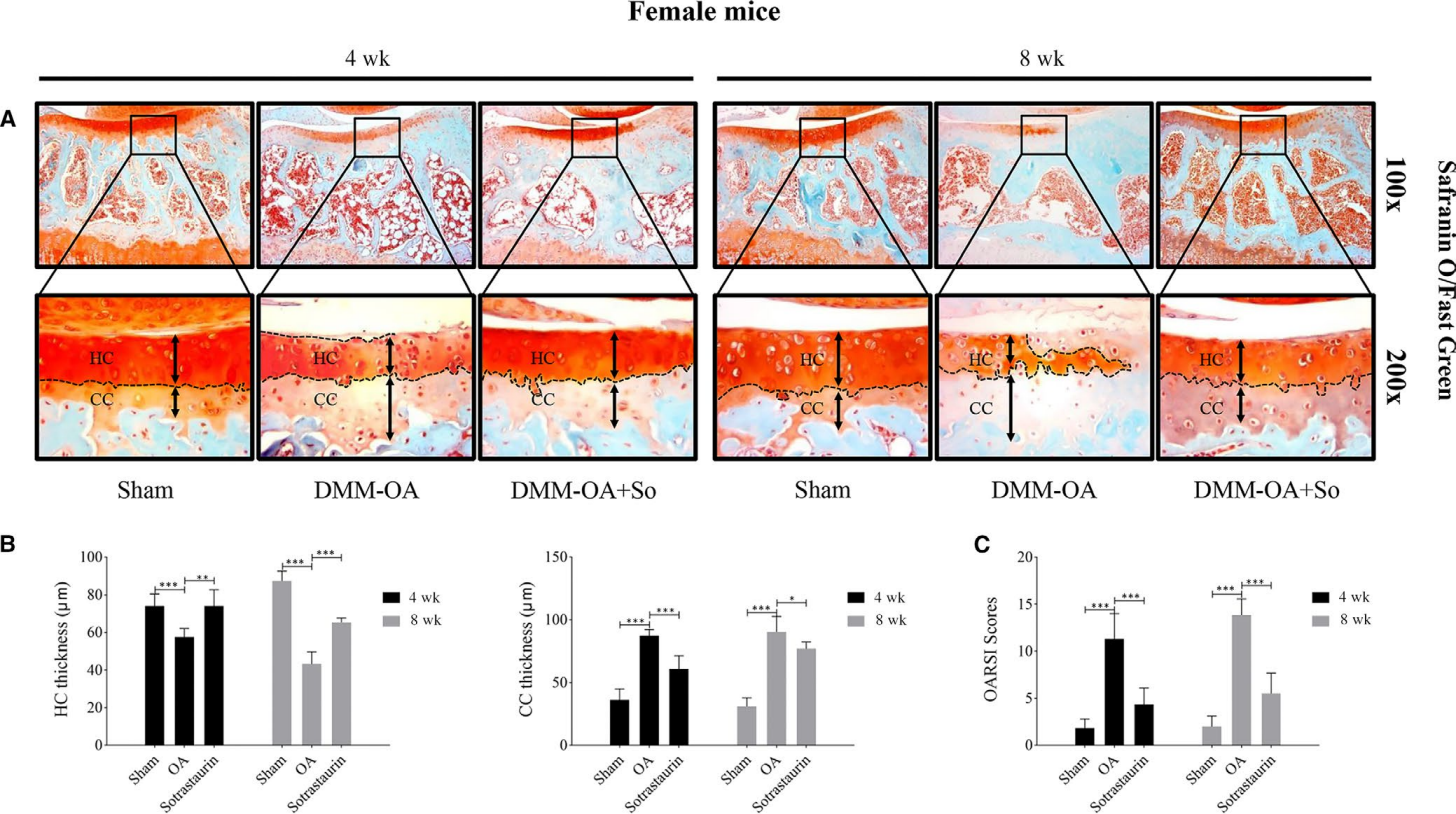
Histological and histomorphometric evaluation of the sotrastaurin effect on cartilage degradation of DMM model in sections. A, Representative images of cartilage stained by Safranin O/Fast green (magnification, 100 × or 200×) from tissue sections of Sham, OA (DMM + PBS) or Sotrastaurin (DMM + Sotrastaurin) groups; The thickness of hyaline cartilage (HC) and calcified cartilage (CC) were marked by dotted line; n = 6 per group. B, HC and CC thickness of articular cartilage. C, Osteoarthritis Research Society International (OARSI)‐modified Mankin scores to cartilage structure damage of knee joint on mice. Data are presented as the mean ± SD (*P < .05, **P < .01 and ***P < .001); SD, standard deviation

We then assessed whether SO exerts any protective effects articular cartilage degeneration following DMM surgery. Cartilage integrity was examined histologically, and the progression of OA was evaluated by the OARSI scoring system. By Safranin o‐Fast Green staining, the articular cartilage of DMM‐OA mice showed significant cartilage erosion, thinning of hyaline cartilage (HC) with pronounced loss of cartilage proteoglycan and increased in calcified cartilage (CC) thickness (Figure 5A,B, female mice; Figure S2a,b, male mice). On the other hand, the severity of cartilage degeneration and destruction in the SO treatment group was much less than the DMM‐OA group, with the articular cartilage surface in the SO treated group exhibiting noticeably more surface regularity. Furthermore, HC thinning and CC thickening were not as pronounced as seen in the DMM‐OA group (Figure 5A,B, female mice; Figure S2a,b, male mice). The histological findings were supported by OARSI scores which were significantly increased in DMM‐OA group and markedly reduced following SO treatment (Figure 5C, female mice; Figure S2c, male mice). Thus, taken together, SO treatment significantly protected DMM‐OA mice against osteoclast‐mediated bone destruction and articular cartilage degeneration and thus possess therapeutic potential in the treatment of OA.

## 4 DISCUSSION

Osteoarthritis (OA) is a prevalent degenerative disease of the joints affecting both articular cartilage and underlying subchondral bone.^7^ The intimate association and crosstalk between articular cartilage and subchondral bone play an important role in the development and progression of OA.^14^, ^27^ It remains as a matter of debate as to which is the precipitating pathology, articular cartilage degeneration or subchondral bone loss. Current therapeutic strategies for OA consist of a combination of pharmacological and non‐pharmacological options that aims to reduce pain and improve joint function. However, most if not all therapeutic options fall short in reversing cartilage degeneration and/or subchondral bone loss, and disease progression thereby undermining the long‐term prognosis of OA. Although considerable advances have been made in understanding the aetiology of OA, the same cannot be said in terms of therapeutic interventions. As such, the development or identification of more effective disease modifying therapies is needed.

In this study, we showed that Sotrastaurin (SO), a novel indolylmaleimide‐based PKCδ inhibitor,^28^, ^29^ inhibited osteoclast formation and bone resorption in vitro via the suppression of RANKL‐induced activation of MAPK signalling cascades. The in vitro anti‐osteoclastic and anti‐resorptive effects of SO were translated into in vivo protective effects against experimental OA induced by surgical destabilization of the medial meniscus (DMM). The DMM surgical model^30^ is a clinically relevant for mimicking chronic OA in mice. The structural and pathological changes in the affected joint closely resemble some of the changes that occur in human OA including subchondral bone loss, cartilage degeneration and joint instability.^30^, ^31^ In our experimental model, DMM‐OA mice treated with SO exhibited markedly reduced subchondral bone loss and articular cartilage degeneration with significantly improved OARSI scores when compared to DMM‐OA mice that received vehicle treatment. The reduced subchondral bone destruction was in essence due to the inhibitory effect of SO osteoclast formation and bone resorption.

Given the extensive crosstalk and interdependence between articular cartilage and the underlying subchondral bone in maintaining the structural integrity of joints, it has become a bit of a paradox as to whether articular cartilage degeneration or subchondral bone loss precipitates the development and progression of OA.^32^ Some researchers believe that the initial changes in the cartilage integrity and microstructure as a result of excessive mechanical stress in OA leads to the release of inflammatory cytokines such as IL‐1β, TNF‐α and IL‐6 that subsequently induce subchondral bone loss.^33^ Often considered as secondary effect, some studies now believe that subchondral bone degradation is a pathogenic trigger in the development and progression of OA with animal studies showing reduced subchondral bone volume and stiffness in the earliest stages of OA.^34^ These initial changes in subchondral bone releases local factors and cytokines that induces subsequent cartilage degeneration. Hence, without the appropriate support conferred by an intact subchondral bone, any attempt to repair or regenerate the overlying degenerative cartilage for hindering OA progression may be futile.^35^ Therefore, targeting subchondral bone loss in OA is an attractive pharmacological intervention in the treatment of OA.

Osteoclasts are the principal cells for bone resorption, and a number of pharmacological agents such as bisphosphonates are widely used in clinical practice to inhibit aberrant osteoclast activity in osteolytic conditions such as osteoporosis, Paget's disease and metastatic bone disease. A number of studies have investigated the potential of bisphosphonate treatment in the management or prevention of OA but with varying degrees of efficacy. For example, Alendronate was found to prevent OA bone pathologies of subchondral bone loss and osteophyte formation as well as articular cartilage degeneration in a chronic OA model in rats.^36^ Similar findings were also seen with Pamidronate which completely inhibited subchondral bone loss and prevented the development of OA in a rabbit model of anterior cruciate ligament transection (ACLT)‐induced OA.^37^ Pamidronate also protected Runx2 transgenic mice (mice with high bone remodelling) from bone loss associated with partial medial meniscectomy‐induced OA.^38^ Moreover, risedronate treatment in patients was found to reduce the level of C‐terminal crosslinking telopeptide of type II collagen (CTX‐II), a marker of cartilage degradation associated with progressive OA, but sustained clinical improvements in disease progression were not observed.^39^ Thus, these studies provide evidence that targeting osteoclast formation and activity to prevent subchondral bone loss to maintain articular cartilage integrity may offer beneficial symptomatic and structural benefits against OA development and progression.^23^

**FIGURE 6:**
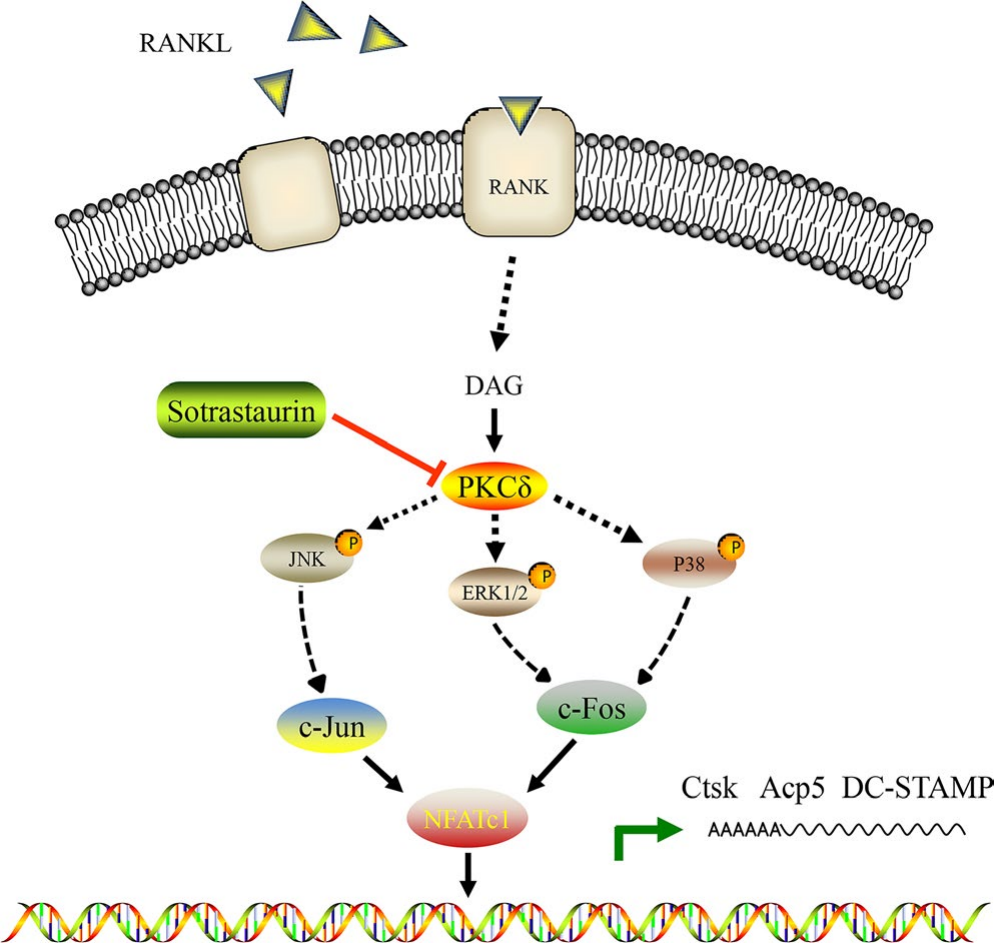
Schematic diagram and potential mechanisms of sotrastaurin inhibition effect on osteoclast resorption. Sotrastaurin inhibits osteoclast resorption activity by inhibiting PKCδ/ MAPKs/ (c‐Fos/c‐Jun)/ NFATc1 signalling pathway

Osteoclasts are multinucleated giant cells derived from the fusion of haematopoietic precursor cells of monocyte/macrophage lineage. The differentiation and fusion of precursor cells are governed by the actions of two key cytokines, M‐CSF and RANKL,^40^, ^41^ which when bound to the respective receptor on precursor cells initiate a cascade of signalling events that lead to the up‐regulation of osteoclast effector genes. The earliest signalling pathways activated by RANKL include the NF‐κB and MAPK signalling pathways, which includes the activation of p38, ERK1/2 and JNK MAPK family members.^42^, ^43^ Recent studies have shown that the protein kinase C family of serine/threonine kinases, particularly the PKCδ which is highly expressed in osteoclasts, as important regulators of osteoclast formation and bone resorption. PKCδ deficiency was found to attenuate the bone resorptive activity of mature osteoclasts in vivo and in vitro via the perturbation in ruffled border formation and inhibition of cathepsin K secretion, a crucial protease for the breakdown of the mineralized bone and cartilage matrix.^20^ Moreover, PKCδ has also been shown to play a role in the upstream regulation of the MAPK signalling pathways.^19^, ^44^ Consistent with these findings, SO was found to inhibit PKCδ expression and suppressed the activation of all three MAPK signalling cascades in response to RANKL. This reduced activation of MAPK signalling consequently hindered the induction of downstream nuclear transcription factors c‐Jun, c‐Fos and NFATc1. NFATc1 is crucial for the transcriptional activation of osteoclast effector genes involved in precursor differentiation and fusion^45^, ^46^, ^47^ (such as *NFATc1* ^48^ and *DC‐STAMP* ^49^), and mature osteoclast bone resorption (such as *TRAP* and *CTSK*). The collective molecular effect of SO resulted in the cellular inhibition of osteoclast formation and bone resorption in vitro (Figure 6). This, in turn, translated to protective effects against DMM‐induced OA in vivo by preventing osteoclast‐mediated subchondral bone destruction as well as reduced articular cartilage degeneration. Our result provided further evidence to suggest the abnormal bone remodelling in subchondral bone may precede cartilage degeneration.

Despite these promising effects of SO on alleviating the osteochondral pathologies of OA, there are still shortcomings in our study and few questions needs to be explored and answered. Firstly, previous study by Khor and colleagues have found that PKCδ‐deficient BMMs exhibited enhanced osteoclast formation potential in vitro despite lower osteoclast numbers in vivo.^19^ However, cause for this discrepancy was not fully determined. Furthermore, our data are in contrast to the findings by Khor and colleagues, where our in vitro cellular assays showed inhibition of RANKL‐induced osteoclast formation in the presence of SO that was associated with reduced gene and protein expression of PKCδ. One likely explanation is that SO is a pan PKC inhibitor and therefore the biological consequence of inhibition of other PKC family members may have had an influence osteoclast formation. The effects of SO on other PKC members will need to be investigated in subsequent studies. Secondly, although we showed that SO inhibits the RANKL‐PKCδ‐MAPK signalling axis, other pathways regulated by PKCδ could also be involved. These include the NF‐κB and PI3K‐Akt pathways which are also crucial for osteoclast differentiation and bone resorption.^50^ The immediate downstream effector(s) of PKCδ needs to be determined to fully comprehend the complex interplay between the multitude of signalling pathways activating during osteoclast formation. Thirdly, in view of the complex crosstalk between bone cells (osteoclasts, osteoblasts and osteocytes) as well as with cartilage chondrocytes, it is necessary to explore the effects of SO on osteogenic functions of osteoblast, the mechanosensory functions of osteocytes particular under altered mechanical load in OA, as well as the chondrogenic functions of chondrocytes. And previous study by Yang et al^51^ have shown that PKC delta KO mice exhibit disorganized chondrocyte morphology, suggesting that PKC delta plays a role in maintaining the normal morphology of chondrocytes; therefore, the specific mechanism of SO protective effects on articular cartilage in histology remains to be further studied. Finally, we cannot rule out possible anti‐inflammatory effects of SO. Inflammation is a crucial aspect for the development and progression of OA, and PKCs have been shown to play important positive roles in the inflammatory response. Thus, it is likely that SO will exert anti‐inflammatory properties that can contribute to the overall protective effects against OA.

In conclusion, our study demonstrated the beneficial role of PKC inhibitor, Sotrastaurin, in the treatment of OA. SO can inhibit RANKL‐induced osteoclast formation and bone resorption in vitro and protects against DMM‐induced OA osteochondral pathologies in vivo. Thus, SO not only prevents subchondral bone loss but also maintain the structural integrity of the overlying articular cartilage which substantially hinders the progression and severity of OA. Together, our data suggest that Sotrastaurin may be further developed as a potential pharmacological agent for the treatment of OA.

## CONFLICT OF INTEREST

The authors have no conflicts of interests to declare.

## AUTHOR CONTRIBUTIONS

LS and PC designed the experiments and drafted the manuscript. PC, WL and LX conducted the in vivo animal surgery and experiments. PC, WL and QH carried out the in vitro cellular and biochemical experiments. PC, WL and ZB analysed and interpreted the results. All authors reviewed the manuscript prior to submission.

## Supporting information

FigS1

FigS2

## Data Availability

The data that support the findings of this experiment are available from the corresponding author upon reasonable request.
